# Toward Accurate and Robust Environmental Surveillance Using Metagenomics

**DOI:** 10.3389/fgene.2021.600111

**Published:** 2021-03-05

**Authors:** Jiaxian Shen, Alexander G. McFarland, Vincent B. Young, Mary K. Hayden, Erica M. Hartmann

**Affiliations:** ^1^Department of Civil and Environmental Engineering, Northwestern University, Evanston, IL, United States; ^2^Division of Infectious Diseases, Department of Internal Medicine, The University of Michigan Medical School, Ann Arbor, MI, United States; ^3^Division of Infectious Diseases, Department of Internal Medicine, Rush University Medical Center, Chicago, IL, United States

**Keywords:** viability, limit of detection, metagenomics, taxonomic resolution, environmental surveillance, quantitative metagenomics

## Abstract

Environmental surveillance is a critical tool for combatting public health threats represented by the global COVID-19 pandemic and the continuous increase of antibiotic resistance in pathogens. With its power to detect entire microbial communities, metagenomics-based methods stand out in addressing the need. However, several hurdles remain to be overcome in order to generate actionable interpretations from metagenomic sequencing data for infection prevention. Conceptually and technically, we focus on viability assessment, taxonomic resolution, and quantitative metagenomics, and discuss their current advancements, necessary precautions and directions to further development. We highlight the importance of building solid conceptual frameworks and identifying rational limits to facilitate the application of techniques. We also propose the usage of internal standards as a promising approach to overcome analytical bottlenecks introduced by low biomass samples and the inherent lack of quantitation in metagenomics. Taken together, we hope this perspective will contribute to bringing accurate and consistent metagenomics-based environmental surveillance to the ground.

## Introduction

Approximately 56% of the world’s population lives in urban areas (United Nations, 2018) and people in developed nations spend at least 90% of the time indoors (Chau et al., 2002; Smith et al., 2016; Cincinelli and Martellini, 2017), making built environments hotspots with which humans frequently interact. Understanding and monitoring fomite transmission is critical in infection prevention (Stephens et al., 2019). The need for environmental surveillance particularly stands out given emerging issues like the COVID-19 pandemic and the continuous increase of antibiotic resistance in pathogens. Metagenomics-based methods have shown promising potential to meet this need, as they can detect entire microbial communities, as opposed to targeted identification.

However, there are several obstacles that we must overcome to bridge the gap between deploying metagenomics and generating actionable interpretations to guide infection prevention. Cultivation provides direct evaluation of microbial removal by revealing observable colonies, but this approach lacks precision (Popovich et al., 2012). Whole genome sequencing leads transmission prevention actions by monitoring strain-level dynamics of the targeted pathogen but is difficult to apply for multiple organisms simultaneously (Deurenberg et al., 2017). Although metagenomics dramatically expands the scope of detectable organisms compared with the aforementioned methods, it urgently needs the ability to differentiate viability, which may otherwise cause an overestimation of infection risk, and reveal the actual load of pathogens (i.e., be quantitative), for direct correlation with infection risk (Valdez et al., 2015; Xiao et al., 2017). Finally, the taxonomic resolution needs to be high enough to discriminate pathogens from closely related non-pathogens.

These challenges are both conceptual and technical in nature. They arise from the diversity of metagenomics research objectives, and are often exacerbated by intrinsic features of low-biomass environments that need to be monitored. Low-biomass samples are typical of built environment surface swabs, air, water, and rocks. Such samples are dilute, containing approximately 10^2^–10^4^ cells/mL for liquid samples (Zhong et al., 2018; Selway et al., 2020). Moreover, these samples are usually limited in total cells, making it harder to obtain enough biomass. For example, when swabbing door handles, the biomass cannot be increased by enlarging the sampling area, which is finite. Thus, special precautions are often necessary due to the low success rate of sample preparation and high possibility of contamination (Eisenhofer et al., 2019).

**FIGURE 1:**
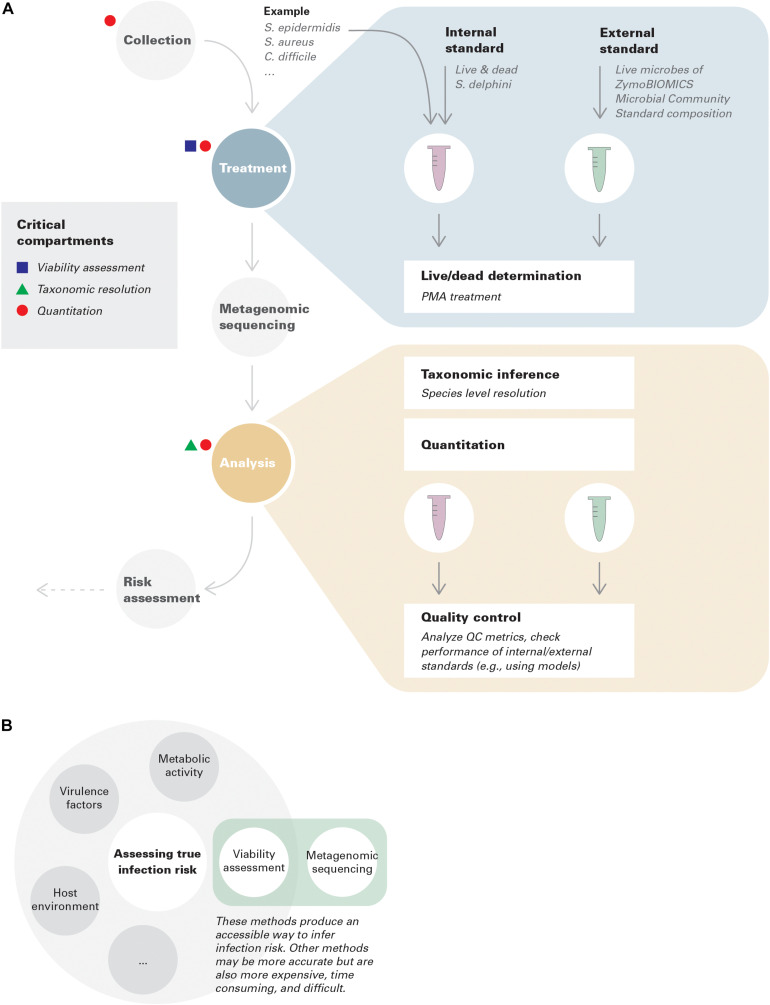
**(A)** Best practices in environmental surveillance using metagenomics (with examples). Internal standards are added to collected samples, while external standards are run in parallel with samples throughout the pipeline to assure its performance. An example is provided for demonstration purposes. Note that the standards given in this example only have theoretical potentials; more investigations are needed for benchmark and optimization. In this example, species level resolution is needed to distinguish *S. epidermidis, S. aureus*, and *S. delphini*. Assuming *S. delphini* is a good internal standard for *Staphylococcus* but not for *Clostridium*, in this case, quantitative risk assessment can only be achieved for *S. epidermidis* and *S. aureus*, but qualitative lesson can still be gained for *C. difficile*. **(B)** Viability assessment coupled with metagenomic sequencing represents an accessible way to infer infection risk.

In this context, we focus on three critical conceptual and technical advances that need to be incorporated throughout the metagenomic environmental surveillance process: viability assessment, taxonomic resolution, and quantitation (Figure 1A).

## Viability Assessment Using Propidium Monoazide (PMA)

Locations of environmental surveillance (e.g., built environment surfaces) harbor a significant proportion of dead microbes, which are captured by traditional DNA-based methods, including metagenomic sequencing (Gomez-Silvan et al., 2018). Failure to assess viability could cause overestimation of infection risk. Approaches have been proposed to address this issue, with PMA treatment as a representative.

PMA treatment directly assesses membrane integrity. However, viability more broadly includes multiple underlying features, such as replication, metabolic activity (Codony et al., 2020), and virulence. These phenomena are not always interchangeable. In environmental surveillance, connecting viability to infection risk is the most informative criterion. This also highlights the importance of clarifying which criteria are being evaluated in the assessment of methods (Figure 1B).

Technical challenges and optimization efforts have accompanied PMA treatment throughout its development (Fittipaldi et al., 2012; Elizaquivel et al., 2014; Emerson et al., 2017; Codony et al., 2020). The outcome is related to multiple factors, including experimental conditions (e.g., dye concentration, incubation time, light exposure time), the diversity of microbes (e.g., target gene length, differences in cell membranes, formation of spores), and the complexity of the matrix (e.g., turbidity, pH, dead cell density). Variations in these factors make PMA treatment seemingly unreliable. It may nevertheless be valuable for environmental surveillance when certain conditions are satisfied.

Application of PMA treatment to environmental samples has been hindered partly because these samples contain a diverse microbial community in a complex matrix. As pointed out by Wang et al., PMA-seq with a universal protocol is not feasible to quantify viability of realistic communities, even with *E. coli* controls spiked in Wang et al. (2020). To facilitate its application in surveillance, instead of insisting on differentiation of the viability for every community member, we should start by identifying sets of similar pathogens, as these groups will have the highest potential to fit in one protocol while maintaining relatively good efficiencies. Comparing this to the concept of pinpointing dynamic range in quantification, by sacrificing part of metagenomics’ randomness, viability quantification may be achieved. For example, Yang et al. have tried to simultaneously detect three viable *Salmonella enterica* serovars using multiplexed PCR coupled with PMA treatment (Yang et al., 2012). Analogous principles have also been applied to the development of reagent enhancers by focusing on Gram-negative bacteria (Codony et al., 2020).

Internal standards help address biases introduced by complex matrices. To that end, peroxide-killed *Campylobacter sputorum* cells were spiked into chicken rinses in the quantification of viable *Campylobacter* (Pacholewicz et al., 2019) with encouraging results. Nevertheless, further progress should be made for widespread adoption of internal standards in risk assessment, particularly regarding the diversity and viable proportion of microbes forming the standard. Internal standards containing more than one organism are necessary to cover the diversity of the targeted microbial group, with different viable proportions to account for variations in PMA efficiency at different live/dead ratios. In routine application, once a stable relative response factor is determined for a microbe (or microbial group)-standard combination, the number of internal standards might be reduced. Creating quality control metrics, analogous to sequencing coverage and depth in metagenomics, or adopting calculation schematics exemplified in Fittipaldi et al. (2011) may also be noteworthy directions for future research.

Briefly, building a well-defined and continuously polished framework that limits its usage to a feasible scope but also maximizes the supporting functionality paves the way toward implementation of PMA treatment in environmental surveillance.

## Inferring Taxonomy in Low-Biomass Metagenomes

Short-read shotgun metagenomic analysis reveals taxonomy without the limitations of amplicon sequencing or culture-based methods (Quince et al., 2017). However, low-biomass samples can be more susceptible to technical factors including library size, community complexity, host DNA, and contamination. Therefore, mitigating strategies should be carefully considered. Afterward, choosing a suitable taxonomic identification method is crucial for reliable metagenome analysis and interpretation, particularly for preventing false over-estimation of pathogens based on detection of non-pathogenic relatives or under-estimation of risk from pathogens with very low infective doses due to limitations in detecting rare taxa.

Differences in coverage and depth can result in differing estimates of taxonomic richness and diversity in identical samples, primarily at low level ranks, such as genus or species (Table 1; Jovel et al., 2016; Zaheer et al., 2018). Smaller read libraries are particularly challenged by a diminished capacity to detect rare taxa and accurately estimate overall taxonomic abundance of samples (Hillmann et al., 2018) because more abundant members in the metagenome have a higher likelihood of detection (Nayfach and Pollard, 2016). Additionally, the lowered overall information content of low coverage and depth read libraries impact the ability to identify low level taxonomic ranks.

**TABLE 1:** Definitions and calculations of common sequencing terms.

Term	Definition	Approach(es)	Reference(s)
Read library	The number of reads generated from a single metagenomics sample	–	–
Coverage	The fraction of a metagenome represented by the read library	Taxonomy-based rarefaction curve Read redundancy-based rarefaction curve	Huson et al., 2007; Rodriguez and Konstantinidis, 2014b
Read depth	The number of times a particular base is captured by a read	*base* × *readsthatmaptobase*	Li et al., 2009; Quinlan and Hall, 2010
Mean read depth	The read depth averaged across the metagenome assembly	$\frac{r\,e\,a\,d\,s\,m\,a\,p\,p\,e\,d\,t\,o\,a\,s\,s\,e\,m\,b\,l\,y\times a\,v\,e\,r\,a\,g\,e\,r\,e\,a\,d\,l\,e\,n\,g\,t\,h}{a\,s\,s\,e\,m\,b\,l\,y\,s\,i\,z\,e}$	Vezzulli et al., 2017

Low-biomass samples are especially sensitive to the presence of contaminants, as the true signal can be easily overwhelmed (Eisenhofer et al., 2019). Multiple avenues of contamination exist, including sample preparation and DNA extraction, from either the reagents or the researchers themselves, and carryover between sequencing runs. Methods to reduce contamination include UV radiation and DNase treatment of kit reagents to specialized library preparation workflows (Tamariz et al., 2006; Silkie et al., 2008; Seitz et al., 2015; Minich et al., 2018). Metagenomic samples should be accompanied with kit extraction negative controls and DNA-spiked positive/internal controls during sequencing runs to identify sources of contamination (Minich et al., 2018; Eisenhofer et al., 2019; McLaren et al., 2019).

Given the susceptibility of low biomass samples to contamination, special care should be taken in preparing appropriate controls to avoid misidentifying contaminants as true signals, as cross-contamination can confound epidemiological or strain-tracking efforts (Lusk, 2014; Lauder et al., 2016). The simplest approach is to remove sample reads that align to taxa found in the negative controls (Breitwieser et al., 2017). This can result in removing reads belonging to the true taxonomic composition of a sample, and especially problematic in instances where negative controls are contaminated by sample DNA or belong to a pathogen under surveillance. Other approaches include filtering sequences that fall below a designated relative abundance threshold or map to taxa in a contaminant database (Davis et al., 2018). Approaches that remove low frequency sequences are not recommended for low biomass samples. Employing blank negative controls and study-specific kit negative controls could help in identifying genuine instances of contamination in low biomass samples and detecting kit-based contamination. Bioinformatics pipelines that incorporate either one or a combination of the above approaches have been developed to streamline identification of contaminants and/or cross-contamination (Schmieder and Edwards, 2011; Davis et al., 2018; Martí, 2019).

A variety of tools are available to characterize the taxonomic composition of a metagenomic sample and broadly follow two approaches: using reads as inputs or assembling reads and then using the genes/contigs as input (Breitwieser et al., 2017; Ye et al., 2019). Both approaches have tools that use k-mer, alignment, and marker gene matching implementations. A meta-analysis of both approaches demonstrated that at artificially lowered read library sizes, read-based classification methods maintained their accuracy compared to assembly based methods (Tamames et al., 2019) because assembly based methods rely on having sufficient overlapping read depths. Metagenomic samples from low-biomass environments with insufficient coverage (<20X read depth over the whole metagenome) may require read-based taxonomic classification (Quince et al., 2017; Tamames et al., 2019). Similarly, inherently low read depths may limit the level of taxonomic resolution, as strain-level analysis requires high read depth to distinguish between SNP variants or marker gene variants (e.g., characterizing the relatedness of strains during an outbreak using SNPs) (Truong et al., 2015; Brito and Alm, 2016; Roe et al., 2016; Hillmann et al., 2018). If strain-level variants are desired, merging paired-end reads or using sequencing technologies that generate longer reads may be necessary (Brito and Alm, 2016).

Choosing an appropriate taxonomic reference database can greatly impact the breadth of taxa identified (Pereira-Marques et al., 2019; McArdle and Kaforou, 2020). For example, a reference database built from gut bacteria may not identify environmental taxa but may be suitable for identifying gut pathogens in the environment. Many tools offer the option of using either precompiled or custom reference databases (Breitwieser et al., 2017). CAMISIM, a tool that simulates microbial metagenomic datasets, can be used by researchers to test different approaches (Fritz et al., 2019).

## Quantitative Metagenomics in Environmental Surveillance

Conceptually, quantitative metagenomics has appeared in many ways in microbiome research, ranging from performing basic calculations of abundance, to normalizating metrics to these calculations, to the ultimate goal of absolute quantification as in qPCR (Pons et al., 2010).

At any level, parameters or metrics for profiling a microbial community are the basis of analysis. As such, selecting meaningful parameters is the first step toward quantitative metagenomics. Of the five parameters summarized in their review (Nayfach and Pollard, 2016; Table 2), Nayfach and Pollard suggested that cellular relative abundance and average genomic copy number are the more biologically meaningful and quantitative strategies. However in reality, relative abundances are more frequently used. For instance, quantitative metagenomics is applied in gut microbiome studies to identify unique biomarkers (Le Chatelier et al., 2013; Wen et al., 2017), to compare disease and health states (Qin et al., 2014), and to predict resistome (Ruppe et al., 2019), all of which use cellular/gene relative abundance normalized by genome/gene length through mapping reads to reference genomes/genes (e.g., the MetaHIT gene catalog).

**TABLE 2:** Parameters to profile microbial communities summarized by Nayfach and Pollard.

Parameter	Theoretical calculation equation
Cellular relative abundance	$C\,R\,A\,\left(i\right)=\frac{n\,u\,m\,b\,e\,r\,o\,f\,c\,e\,l\,l\,s\,o\,f\,t\,a\,x\,o\,n\,i}{n\,u\,m\,b\,e\,r\,o\,f\,c\,e\,l\,l\,s\,i\,n\,t\,h\,e\,c\,o\,m\,m\,u\,n\,i\,t\,y}$
Gene relative abundance	$G\,R\,A\,\left(i\right)=\frac{n\,u\,m\,b\,e\,r\,o\,f\,g\,e\,n\,e\,i}{n\,u\,m\,b\,e\,r\,o\,f\,g\,e\,n\,e\,s\,i\,n\,t\,h\,e\,c\,o\,m\,m\,u\,n\,i\,t\,y}$
Average genomic copy number	$A\,G\,C\,N\,\left(i\right)=\frac{n\,u\,m\,b\,e\,r\,o\,f\,g\,e\,n\,e\,i}{n\,u\,m\,b\,e\,r\,o\,f\,c\,e\,l\,l\,s\,i\,n\,t\,h\,e\,c\,o\,m\,m\,u\,n\,i\,t\,y}$ *AGCN*(*i*) = *GRA*(*i*) × *average genome size* (*in number of genes*)
Cellular absolute abundance	$C\,A\,A\,\left(i\right)=\frac{n\,u\,m\,b\,e\,r\,o\,f\,c\,e\,l\,l\,s\,o\,f\,t\,a\,x\,o\,n\,i}{v\,o\,l\,u\,m\,e/w\,e\,i\,g\,h\,t/a\,r\,e\,a}$
Gene absolute abundance	$G\,A\,A\,\left(i\right)=\frac{n\,u\,m\,b\,e\,r\,o\,f\,g\,e\,n\,e\,i}{v\,o\,l\,u\,m\,e/w\,e\,i\,g\,h\,t/a\,r\,e\,a}$

**Theoretical calculation equations are created based on our interpretation of the authors’ descriptions, which are also adapted to the context of environmental surveillance.**

However, cellular and gene absolute abundances are the most promising parameters in environmental surveillance, which is predicated on the actual load of pathogens or pathogenic genes. Moreover, absolute abundances allow better comparisons across samples (Satinsky et al., 2013) and across taxa/genes (Frank and Sørensen, 2011; Nayfach and Pollard, 2016).

Technically, several challenges remain to be overcome toward accurate and unbiased estimation of absolute abundances. It requires careful re-design of the entire study in a stringently quantitative framework, beginning with sample collection. Samples should be collected in an absolute framework (per unit volume, weight, area, etc.), and this framework should be maintained throughout sample preparation. Taking surface microbiome studies as an example, extra considerations include the measurement and documentation of the swabbing area as well as volume of sampling buffer and other solutions used in the entire workflow, and the examination of the recovery rate where sample loss is non-negligible. Furthermore, normalization by genome/gene length is necessary to account for the varying representativity in sequencing a read from genomes/genes of different lengths.

This is not always easy in reality. For example, the sampling area of sink biofilms is difficult to assess when destructive sampling is not permitted. Even if the samples are collected in a strictly quantitative way, other steps in the sample treatment process still need to be conducted quantitatively. Taking DNA extraction as an example, instructions like “transfer up to 600 μL of supernatant to a clean tube” destroy the quantitative chain and prevent us from calculating dilution factors. Accurate normalization by genome/gene length requires continuous effort in expanding genome/gene databases and in incorporating genome/gene normalization into bioinformatic pipelines (Kerepesi and Grolmusz, 2016). In the interim, mapping reads to a set of well-studied while also universal (within the study scope) marker genes (e.g., 16S rRNA genes in bacteria) could serve as a workaround (Nayfach and Pollard, 2016) but unfortunately introduces its own biases.

Besides incorporating qPCR or flow cytometry, introducing standards has great potential to enhance the quantitation ability of metagenomics. In this context, internal standards outcompete external standards, partly because variations in sample treatment seem inevitable (e.g., shotgun libraries undergo equimolar normalization) and because the relationship between the amount of input material and the number of output reads remains obscure. Internal standards also compensate for errors resulting from any non-quantitative processing steps following their addition. Some efforts have been made to incorporate internal standards into the metagenomic pipeline, such as spiking mock-community cells into the collected samples (McLaren et al., 2019), adding genomic DNA just prior to cell lysis in DNA isolation (Satinsky et al., 2013, 2014), and including a set of synthetic DNA before library preparation (Blackburn et al., 2019). Despite these advances, systematic investigations are needed to benchmark methods, identify limitations, and validate use in various contexts. Clearly, a set of standards are needed to account for the complexity of samples and the diversity of targets. But which performs the best among mock communities, genomic DNA isolated from cultured microorganisms, and synthetic DNA remains unclear. Nor is it known at which step the standards should be added and at what dose. Moreover, when exogenous materials are hard to find, the standard addition method may be worth exploring (Danzer, 2007). Finally, the standards should be evaluated holistically at the pipeline level for their compatibility and functionality across multiple steps (e.g., PMA treatment, metagenomic sequencing). Ideally, an optimal pipeline should also be equipped with quality control compartments such as external standards and mathematical models which assess and calibrate biases (McLaren et al., 2019).

## Limits of Detection

Incorporating viability assessment, adequate taxonomic resolution, and quantitation into metagenomics will yield invaluable insights into environmental surveillance. But perhaps more critical than interpreting observed data is interpreting non-detects. Ultimately, a viable signal must be linked to infection risk by determining the threshold load of pathogens to cause an infection when they are contracted from a fomite. This threshold is pathogen-specific. Thus, reference values, like clinical standards for antimicrobial susceptibility testing (CLSI, 2018), as technological standards are necessary.

Moreover, sensitivity of every step in the pipeline must be accounted for in data interpretation, as the overall sensitivity is determined by the lowest step. As mentioned above, features of the community being sampled can affect the expected breadth of coverage. Complex samples with many taxa, host DNA, or stochastic eukaryotic DNA (for example in surface swabs) may require high read library sizes to ensure sufficient breadth of coverage (Ballenghien et al., 2017; Pereira-Marques et al., 2019). This is especially important when non-target DNA can represent the majority of the reads generated, resulting in decreased capacity to detect rare taxa and at fine-grained resolutions. Pilot studies that assess coverage using taxonomy or read redundancy-based rarefaction analysis can help determine an appropriate library size (Rodriguez and Konstantinidis, 2014a). When pathogens are rare compared to other organisms, limit of detection (LoD) is a crucial parameter as it determines the maximum possible load of pathogens when they are not detected. Given the inherent nature of metagenomic shotgun sequencing that a fixed total number of reads are distributed based on the relative proportion of genetic materials present in a batch, LoD must be approximated with the microbial community to be sequenced at a batch-based pace. Because of this matrix-dependent characteristic, it is impractical to get a universal LoD for the technique “metagenomic sequencing.” Empirically, LoD can be estimated relative to the least abundant but detected members in the internal standards or the sample itself.

## Discussion

In summary, metagenomics has enormous potential in environmental surveillance of pathogens as it simultaneously detects multiple organisms and functional genes of interest, e.g., antibiotic resistance. However, the following steps need to be taken to ensure that metagenomic data can practically be applied to risk assessment:

1. Rationally address inherent conceptual limitations regarding viability. For example, PMA treatment assesses membrane integrity, not infectivity; but relationships can be deduced between intact cells and infectious organisms.
2. Rationally address inherent limitations regarding taxonomy. For example, while almost all *Salmonella* are pathogenic, higher taxonomic resolution is needed to distinguish pathogenic *Pseudomonas*.
3. Incorporate internal standards. Doing so will compensate for biases introduced by complex environmental matrices, yield quantitative results, and correct both random and systematic errors.
4. Holistically integrate multiple steps in pipeline optimization. Specifically, internal standards can be incorporated for multiple operations including PMA treatment, taxonomic inference, and quantification.

Metagenomics-based environmental surveillance has potential for developing rich datasets that aid surveillance. Metagenomic data can aid in linking taxa with virulence factors and antibiotic resistance genes. Strain-level data can further track transport of pathogens in the environment or reveal microbial networks of interactions among patients, employees, medical devices or wastewater. Promoting crucial standardizations ranging from sampling protocols to data analysis, curation and presentation, cannot only help produce internally consistent results but also increase external compatibility with data generated in different studies (Nayfach and Pollard, 2016) or with different protocols (Sinha et al., 2017).

## Data Availability Statement

The original contributions presented in the study are included in the article/supplementary material, further inquiries can be directed to the corresponding author/s.

## Author Contributions

JS managed the project. JS and AM co-wrote the manuscript. EH, VY, and MH provided editorial input. All authors have read, edited, and approved of the final manuscript.

## Conflict of Interest

The authors declare that the research was conducted in the absence of any commercial or financial relationships that could be construed as a potential conflict of interest.
